# The Willing-Unwilling: A Cross-Sectional Study to Explore Health Workers’ Perceptions About the COVID-19 Vaccine

**DOI:** 10.7759/cureus.92665

**Published:** 2025-09-18

**Authors:** Khurram Shahzad, Zaeem Ul Haq, Assad Hafeez

**Affiliations:** 1 Epidemiology and Biostatistics, Health Services Academy, Islamabad, PAK; 2 Immunization, Gavi, the Vaccine Alliance, Islamabad, PAK; 3 Epidemiology and Public Health, Health Services Academy, Islamabad, PAK

**Keywords:** communication strategy, covid-19, health worker, trust, vaccine acceptance, vaccine hesitancy

## Abstract

Background

Health workers (HWs) are considered the most influential promoters of immunization. This notion, however, was challenged during COVID-19 when health workers in several countries asked for more information before supporting mass vaccination. In Pakistan, many health workers registered in the system but were delaying their vaccination during the early days. We conducted this study to assess the acceptance of the COVID-19 vaccine along with the reasons for delays or refusals among health workers in Pakistan.

Methods

From a pool of 20,000 health workers registered to receive the vaccine, we called 500 randomly selected health workers (doctors, nurses, community health workers, pharmacists) via phone. Overwhelmed by their work during the pandemic, only 234 could complete the survey. The survey was done in the early weeks of the COVID-19 vaccine rollout (February-March 2021) to inform the policy.

Results

About 3/4^th^ of participants although willing, but were yet to vaccinate because 69 (39%) were busy in clinical duties, 28 (16%) were wary of its use in pregnancy or lactation, 23 (14%) had not received the text message with logistic details, 14 (8%) had a long distance from their vaccination center, 12 (7%) were currently having COVID-19 infection, while remaining 15-20% had a co-morbidity, were currently suspecting COVID-19 infection, or had just recovered from it. Chi-square tests did not reveal any significant association between gender and job designation with willingness.

Conclusion

Health workers have a high acceptance of new vaccines, yet policymakers must not expect that health workers will always be uniformly supportive of new vaccines. Health system strategies must include an ongoing process of documenting the information needs, questions, and apprehensions of health workers and addressing them in real time.

## Introduction

Outbreaks are inevitable, with a chance that some might turn into a pandemic. Public health experts recommend vaccines as a critical response measure to curb the outbreaks. However, it is natural for the public to ask questions about these measures, especially the vaccines, before their adoption [1]. Health workers (HWs), including doctors, nurses, pharmacists, paramedics, and community health workers, are considered an important pillar of communication strategies for responding to such queries [2]. Beyond the pandemic of COVID-19, new vaccines like the human papillomavirus (HPV) vaccine - a critical intervention to reduce cervical cancer and save the lives of women also require strong advocacy and communication by these HWs. Women and girls living in patriarchal settings, where it is commonly believed that vaccines given to young girls cause infertility, have often found a health worker's counselling very helpful [3]. 

Today, aside from using new vaccines to respond to pandemics, routine vaccines for children-that saved an estimated 146 million lives during the past 50 years- also need special attention. According to recent estimates of national immunization coverage by the WHO and UNICEF, childhood immunization, which stalled during the COVID-19 pandemic, is yet to regain its pre-pandemic position. There are 21 million un- and under-immunized children, 2.7 million more than in 2019 [4]. In addition to disrupted systems, hesitant caregivers are also responsible for these numbers, as they often have concerns about vaccines. The existing models for improving vaccine uptake put HWs at the heart of addressing the caregiver concerns because communities have consistently shown confidence in them. Some data even suggest that perceptions and practices of HWs about vaccines have a correlation with vaccination among the populations served by them [5]. 

Amid the pandemic of COVID-19, however, the view that HWs would always support vaccine uptake was shaken. Policymakers and managers in several countries realized that HWs may also have critical questions to ask about the vaccine. For example, HWs in France and French-speaking areas of Belgium and Canada were 80% positive about recommending the COVID-19 vaccine to their population, but only 72% willing to receive it themselves [6]. In a multi-country study (14 European and 1 African), about 1/3rd of the HWs were unwilling to vaccinate as they were not satisfied with the vaccine information provided by the authorities [7].

Pakistan, a lower-middle-income country with a massive population of 250 million, also had similar challenges. The government introduced the COVID-19 vaccine in February 2021 [8]. A digital database called National Immunization Management System (NIMS) was created under the auspices of the National Command and Operations Center (NCOC), the highest office for strategic planning on COVID-19 in Pakistan. Through media and public announcements, people were asked to register and were later contacted by the system with the information about their date, time, and place of vaccination [9]. Owing to supply issues and the required prioritization, HWs were the first recipients of this vaccine. Their vaccination was critical, as they were on the frontlines. Moreover, it was also important to improve acceptability among the broader population. However, it was observed in the initial weeks that many HWs were not getting vaccinated. 

Compounding the problem were the anecdote-laced stories and opinion articles in the media. Without exploring the real local reasons for the so-called negative perceptions, the media were publishing misleading stories about health workers' reluctance to receive the vaccine [10]. What was the scale of reluctance, and why these HWs were not receiving their vaccine, was an important question for the health workers’ protection, and the overall pandemic response. Moreover, it had obvious implications for the long-term vaccination policies and strategies. We conducted a rapid study to assess the acceptance of the COVID-19 vaccine, along with reasons for delays or refusals among health workers in Pakistan. 

## Materials and methods

Study design and setting 

This was a cross-sectional, descriptive study to assess the acceptance of the COVID-19 vaccine and the reasons for refusal among the health workers in Pakistan. The study was conducted very early during the vaccine deployment in the country, a time when real-world information about the vaccine’s acceptance was minimal. One-time, cross-sectional surveys are considered appropriate for such studies that aim to quickly understand a naturally occurring phenomenon in emergency conditions [11]. For this study, we adopted a phone-based data collection method to meet the urgent need for evidence and to avoid the risk of virus transmission.

Population and sample size

The inclusion criteria for participants in this study included healthcare workers of all types (doctors, nurses, pharmacists, paramedics) from all sectors (public, private, NGO) residing in any part of the country who had registered with NIMS but had not yet received the first dose of the vaccine. The NIMS records showed that over 20,000 health workers registered in the database but had not received the vaccine till that time. This segment was the population for our study. Anticipating that at least 70% will show willingness, keeping a 95% confidence level, and 5% margin of error, we calculated that 323 participants will be required for this study. To enroll them, we used a computer system to generate random numbers from NIMS and selected 500 individuals to be invited to this study. 

Data collection and analysis

Before COVID-19, most survey instruments for assessing vaccine acceptance - including those recommended by the WHO's Strategic Advisory Group of Experts (SAGE) - pertained to child vaccination [12]. Since our study aimed to explore adults' willingness regarding their vaccination, we developed a new tool for this study. This short, structured questionnaire (Appendix) had three sections. Sections one and two dealt with demographics and the recent history of COVID-19, respectively. Section three drew upon the three attitudinal concepts about vaccine acceptance, including convenience, complacency and confidence [13] and inquired about reasons of not having received the vaccine, using the following response items: i) did not have time to visit vaccinator center ii) want to delay till sufficient data on safety is available and iii) do not want to receive vaccine because of a) fear of adverse effects b) not convinced about the efficacy c) other reasons. 

Trained enumerators collected the data by making phone calls. Each randomly selected participant was called at least three times if the first attempt at the interview failed. Failure to collect data, even after three calls, was recorded as no-response. Only the fully completed calls were considered fit for inclusion in the study for final analysis. For the COVID-19 vaccination, we inquired about willingness, followed by the reason for not having received the vaccine yet. Reasons for not intending to receive the vaccine were also asked if the respondent expressed unwillingness. Although we did not solicit any qualitative observations, some participants shared their views without prompting, which were also noted. 

The study was conducted during the early phase of the vaccination drive for COVID-19 in Pakistan (25th February-6th March 2021). In total, we collected data from 234 HWs in the time available to complete the data collection. Descriptive analysis was performed to estimate acceptance rates by gender, age, job designation, and years of experience. Chi-squared test was performed to assess any meaningful relationship between the categorical variables and willingness to receive the vaccine [14]. Our methodology acknowledged that individuals in some geographical areas may not be represented due to a lack of tele-density in these areas. 

Ethical considerations 

Participants were briefly informed about the survey through interactive voice response (IVR), and their response in IVR was considered as consent. Risks and benefits were explained to the participants. The survey posed minimal risk as it was a telephonic interview, not requiring physical interaction with enumerators or any administration of tests. The benefit to the participants was indirect, as the information they provided would help the federal and provincial governments to improve their plans. Participant confidentiality was maintained throughout the study. All subjects who participated in the investigation were assigned a study identification number. The study was cleared (No. 7-82/IERC-HSA/2021-12A) by the Ethics Committee of the Health Services Academy, Islamabad, under the rapid review process for those COVID-19 studies that ensured participant anonymity and did not involve biomedical samples or physical interaction.

## Results

We collected data from 234 participants. With the country undergoing another peak of COVID-19 cases in March 2021, health workers were extremely busy and required repeated calls to complete an interview. On the other hand, the NCOC needed the information quickly to make critical decisions about the COVID-19 vaccination in the country. To appraise the policymakers, findings from 2/3rd of the sample were presented to the NCOC. The NCOC reached some immediate decisions (e.g., making vaccines mandatory) that were quickly disseminated through the media [15]. After this, the participant's response could be biased about their willingness or unwillingness in the context this policy environment. It was, therefore, decided to stop further data collection and use the available numbers for analysis. 

In all, 234 HCWs from Pakistan-including doctors, nurses, paramedics, and support staff-participated in this study. Descriptive statistics for the participant demographics and their association with vaccine acceptance are presented below. Among those who completed their interviews (Table 1), 145 (62%) were male and 89 (38%) were female. Professionally, 54 (23%) were doctors, 33 (14%) nurses, 24 (10%) ancillary staff, while 80 (34%) belonged to the category of others. The mean years of experience was 7.28 years (SD 6.72), while 3/4th of the participants were in the early part (0-10 years’ experience) of their career. 

**Table 1:** Demographic characteristics and willingness to receive the vaccine (n=234)

Characteristic	Description	Willing to receive the COVID-19 vaccine		Statistic* and p-value**
		Yes, N=177 (76%)	No, N=57 (24%)	
Gender				
Male	145 (62%)	115 (79%)	30 (21%)	χ² = 2.79, p = 0.095
Female	89 (38%)	62 (70%)	27 (30%)	
Age				
18-30 years	117 (50%)	96 (82%)	21 (18%)	χ² = 5.23, p = 0.073
31-50 years	98 (42%)	68 (69%)	30 (31%)	
>50 years	19 (8%)	13 (68%)	6 (32%)	
Professional category				
Doctor	54 (23%)	38 (70%)	16 (30%)	χ² = 7.09, p = 0.312
Nurse	33 (14%)	23 (70%)	10 (30%)	
Lab technician	15 (6%)	10 (67%)	5 (33%)	
Paramedic	14 (6%)	9 (64%)	5 (36%)	
Polio worker	14 (6%)	10 (71%)	4 (29%)	
Ancillary staff	24 (10%)	21(87.5%)	3 (12.5%)	
Other	80 (34%)	66 (82.5%)	14 (17.5%)	
Years of experience				
0-10 years	173 (74%)	128 (74%)	45 (26%)	χ² = 2.71, p = 0.258
11-20 years	41 (18%)	35 (85%)	6 (15%)	
>20 years	20 (8%)	14 (70%)	6 (30%)	
*Pearson's Chi-squared test. **Significance level at p < 0.05.				

Overall, 177 (76%) respondents were willing to receive the vaccine (Table 1) but had not initiated because of one or more reasons. The remaining 57 (24%) were unwilling to receive the vaccine at the time of this study. The results of the chi-square test to find the association of categorical variables, including age, gender, professional category, and length of experience with willingness to receive the vaccine, are presented (Table 1) below. There was no statistically significant association between gender and willingness to vaccinate (χ² = 2.79, df = 1, p = 0.095), age and willingness (χ² = 5.23, df = 2, p = 0.073), job designation and willingness (χ² = 7.09, df = 6, p = 0.312), and between years of experience and willingness (χ² = 2.71, df = 2, p = 0.258).

**Figure 1:**
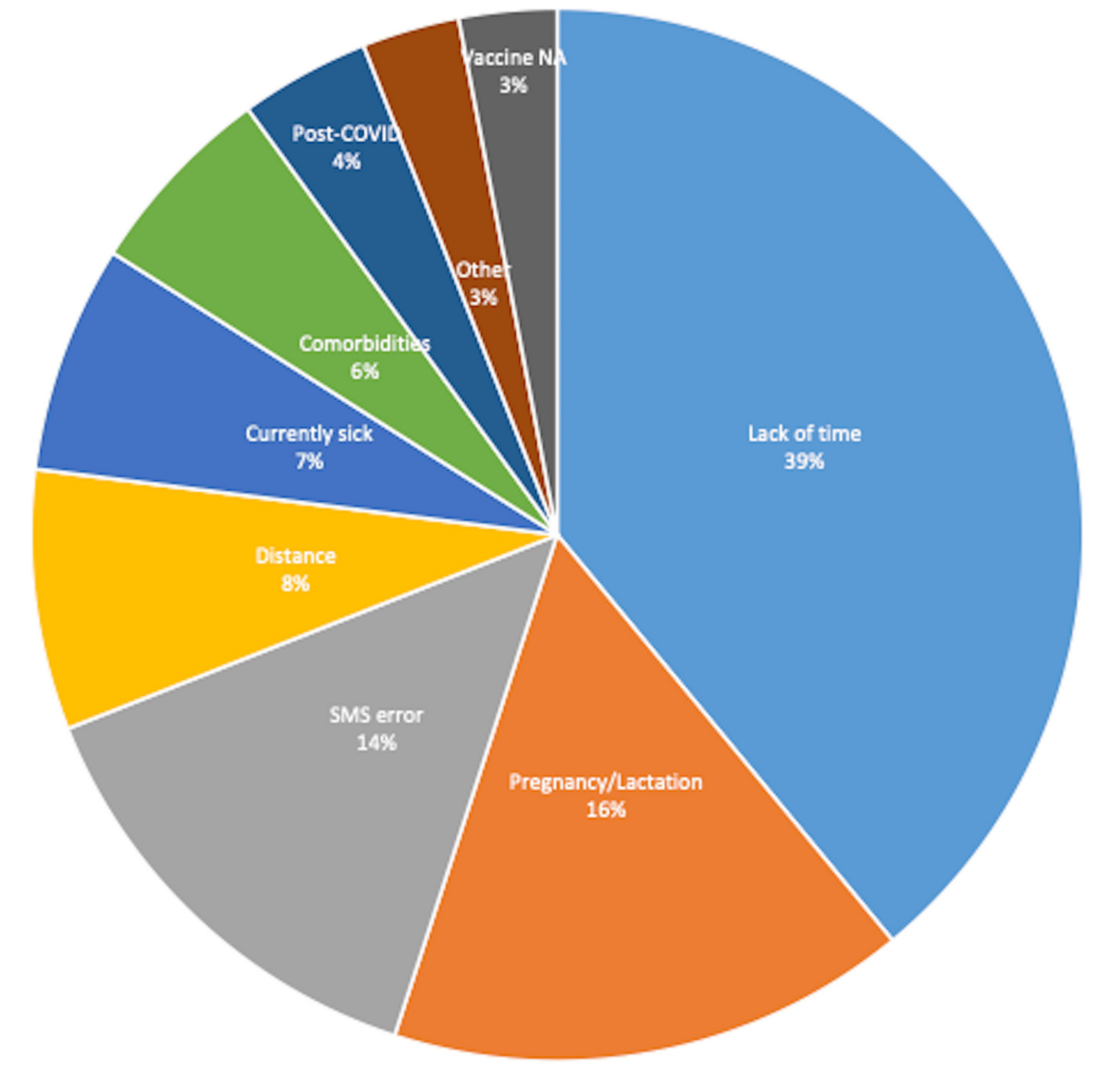
Reasons for not receiving the vaccine despite registering with the system (n=177)

The 177 (76%) study participants who were willing but were not yet vaccinated shared several reasons (Figure 1) for their delay. Among them, 69 (39%) did not have time because of their clinical duties. Another 28 (16%) were contemplative because of pregnancy or breastfeeding their baby. Technology was coming in the way of 23 (14%), as the short text messages about the vaccine date, time, and place reaching them had errors. The inaccessibility of the vaccination centre because of distance for 14 (8%), currently having COVID-19 for 12 (7%), having co-morbidities for 11 (6%), being in immediate post-COVID condition for 7 (4%), and non-availability of the vaccine for 6 (3%) were other reported reasons.

**Figure 2:**
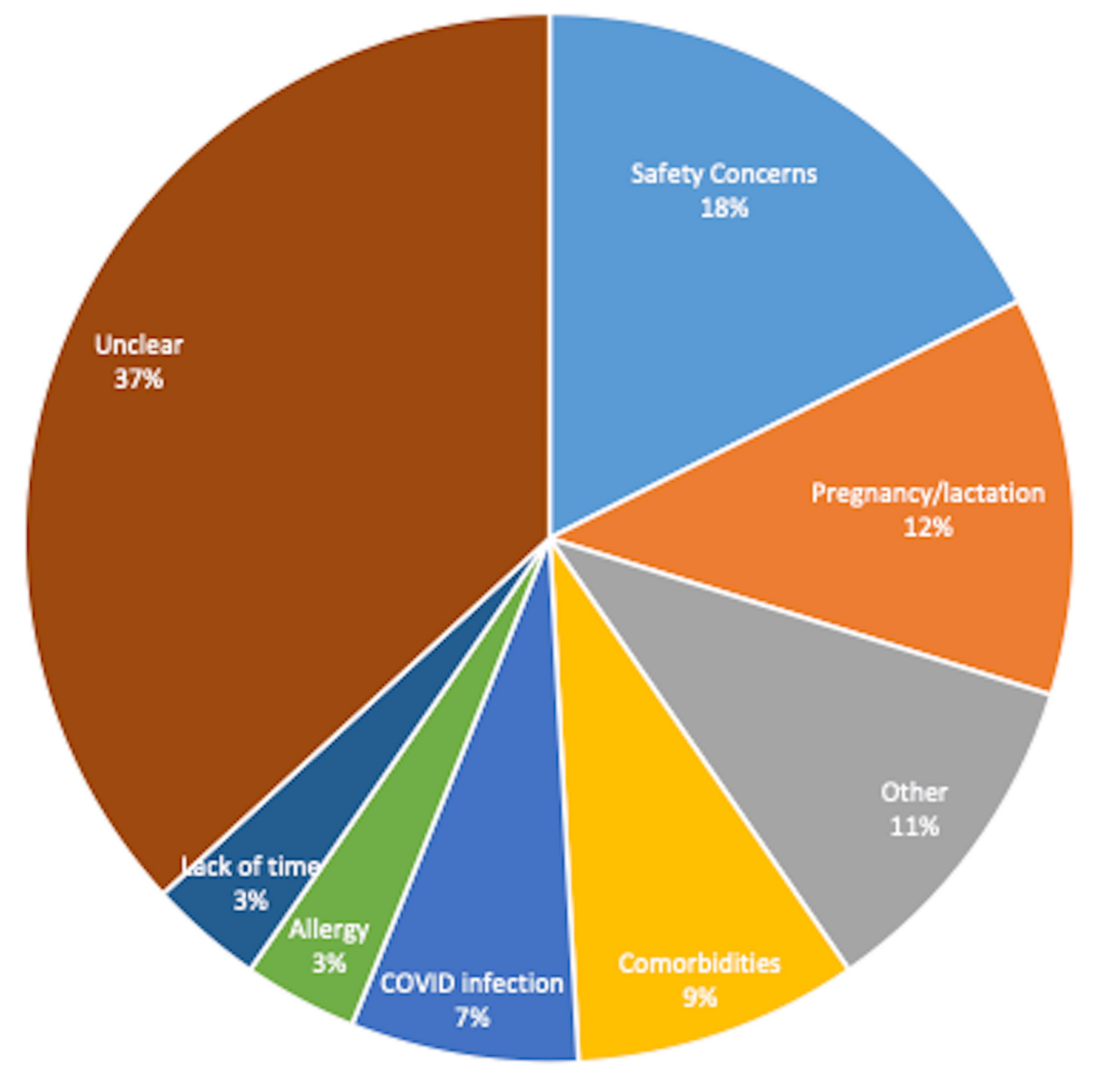
Reasons for refusing the COVID-19 vaccine (n=57)

About a quarter of the participants shared that they were not intending to get vaccinated. Among them, 36 (63%) shared reasons for this decision, while 21 (37%) did not have a clear reason to share. Among those who did share the reason (Figure 2), 10 (18%) had concerns about the vaccine safety, 7 (12%) did not want it because of pregnancy or lactation, and 5 (9%) had comorbidities, 4 (7%) reported they just had COVID-19 infection, while the remaining shared allergy, lack of time, or other miscellaneous reasons for their decision.

Although we did not solicit qualitative insights, we did record observations if a study participant wished to share. One insight that several participants shared was,

“It (the campaign) has started by vaccinating all the frontline healthcare workers, and we have been told this step was essential to increase vaccine acceptance and reduce hesitancy among the people. But what about our questions? Where should we raise them, and who will respond?”

Some were wary of the past year's COVID-19 experiences, which had negatively impacted their trust in the system. One participant said,

“For a full month, I was given 10 masks and a small bottle of sanitizer, with instructions to make them last for the whole month. It was up to me whether I used it all in one day or stretched it over a full month. Even the masks we received were basic. They were not N95, and the prices of masks in the market had increased tenfold."

Rapidly evolving guidelines were another frequent concern.

“We do not have a clear answer to questions like whether pregnant and lactating mothers must receive the vaccine or not, or whether different vaccines being used have the same efficacy or not. Decisions are quickly changed without giving a good enough reason.”

Availability of the vaccine and maintenance of its cold chain were other concerns.

“My concern is that the vaccine is still not available, and my visit to the vaccination centre will just be a waste of time. I keep hearing from government colleagues about dose shortages and bad cold chain maintenance. With frequent power outages, we know the reality of storage conditions and the cold chain at government facilities. So, why get a shot that is equal to nothing?"

## Discussion

This quick study was conducted against the backdrop of unexpected delays by HWs in receiving the COVID-19 vaccine. The results showed that about 3/4th of the participants were willing to vaccinate but had not received it as yet. In addition to concerns about the vaccine, important logistical barriers were identified, which hindered its slow adoption. Our study also revealed that about a quarter of participants were reluctant to receive the vaccine. Some of their concerns overlapped with the concerns of willing (e.g., pregnancy or lactation, COVID-19 infection), but HWs in this category were also reluctant due to safety concerns, unclear guidelines for those having COVID-19 infection, or other comorbidities.

A majority in our study was willing to vaccinate, a finding corroborated by several subsequent studies from Pakistan. In their research with 5237 HWs from across Pakistan during Dec 2020-Feb 2021, Malik et al. found 70% willing to receive, 25% wanting to delay till more data was available, while 5% outrightly rejected the vaccination [16]. Researchers of a hospital-based study in the city of Karachi found that 83% of the 331 subjects were vaccinated in May-June 2021, 15% wanted to delay, and 2.4% were outright refusers. Safety and efficacy of the vaccine were the biggest concerns for the hesitant participants [17]. How many of the vaccinated HWs received it because of the pressure of vaccine mandates, as has been reported from elsewhere, is not clear [18]. 

HWs are an influential segment of society that can facilitate decisions of their community; therefore, a quarter of participants who were undecided in our study cannot be ignored for the following reasons. Number one, owing to their technical background, HWs can have profound questions about vaccines, which must be anticipated and responded to. The high uncertainty about the long-term safety of vaccines and efficacy against mutant strains can make it difficult for HWs to balance the benefits against the risks, as it did in the U.K. [19]. Fears about side effects, lack of trust due to a rapid development of the vaccine, and feeling pressured to accept vaccine mandates also act as impediments [20]. That about a quarter of studies in a systematic review reported HW apprehensions to COVID-19 vaccination, as they did in our research, is enough to realize that routine information is not adequate to address the concerns of health workers, and that more should be done [21]. 

Number two, addressing their concerns improves the trust of HWs in the system, which is critical for the success of the response to a health emergency [2]. In Italy, knowledge of disease and vaccine had a positive correlation with attitudes: HW's intention to vaccinate increased by 75% from Dec 2019 to Dec 2020, as their knowledge about vaccine-preventable diseases improved [22]. In Israel, HWs regarded information from medical sources as their greatest influence on health-related decisions, while the public had a high trust in the health-care providers, both predicting a lower perceived risk toward the vaccine [5]. Quite naturally, therefore, systematic reviews have recommended addressing the HW concerns, as their hesitancy can affect vaccination attitudes among populations [23].

Number three, like the public, the perceptions of HWs about vaccines may also waver over time. About 3/4th of the HWs in our study were positive towards COVID-19 vaccination but had not received it because of being on a spectrum of decision making [24]. Data from the U.S. also support this concept, as the HW tendency to recommend the COVID-19 vaccine declined over time. Their trust in the CDC, which also declined over time, directly correlated with their recommendation, while their dislike of the vaccine mandates was inversely related to recommending the COVID-19 vaccine [25]. 

The above findings draw our attention to a critical gap in the current guidelines for vaccine promotion that emphasize HW training and capacity building [26], implying that an HW is “pro-vaccine” by default. The guidance ignores the demographic (age, race, place of living) and psychographic (the way they think and make decisions) characteristics of HWs that may have a bearing on their own perceptions [27]. Scientists emphasize equipping physicians and nurses with information that helps people [28]. The popular, 7 ‘Cs’ model to improve vaccine acceptance mentions the role of HWs in building ‘confidence’, and presses upon increasing HW knowledge, their interpersonal communication, and debunking skills [29], glossing over the critical questions and concerns the HWs may have, that must be addressed. 

Some limitations must be noted for a realistic interpretation of this study. One, this was a one-time data collection in which the participants shared their thoughts and reasons about their vaccination. Whether they continued to have these perceptions or changed cannot be interpreted from this data and must be gauged through follow-up studies. Two, it was self-reported data, and the subjective self-evaluation and reporting may not reflect the true picture of their attitudes and behaviors. Three, it was a telephone-based survey, and individuals in some geographical areas may not be represented due to a lack of tele-density in their areas. Four, we could not collect data from the full sample calculated for this study because of systemic pressure and time sensitivity that researchers frequently faced during this emergency [30]. Finally, we used a short survey given the time pressures, and while this tool may be useful in emergency settings, it may not serve the purpose of assessing vaccine acceptance in normal settings. Yet, the study has merit because of its unique willing-unwilling mix of participants and alignment of findings with studies conducted in other countries.

The study reinforces the suggestion that HWs may not always be “pro-vaccine” by default and may need sensitization and training through tailored approaches. The approaches can include fulfilling their information needs, responding to questions, and addressing apprehensions about a new vaccine or an existing vaccine. The HW’s perceptions and practices about a new vaccine or repeated campaigns involving existing vaccines must be gauged periodically and addressed through refresher training. Continued engagement with HWs must also be maintained to know their capacities and help them build their skills as influencers. Modifying communication strategies based on concerns shown by health workers can also minimize vaccine hesitancy among people, and must be part of the public policy.

## Conclusions

Health workers are the best platform for a two-way dialogue between people and the health system. However, they can fulfill this role if their own questions and concerns are prioritized and responded to. Our study, like several others from the COVID-19 times, suggests that not all HWs will be pro-vaccine by default. They can have genuine questions that must be answered. Given the results of our study and keeping the current trends of misinformation about vaccines in context, a sustained process of gauging the vaccine concerns among health workers must be an integral part of health systems. The vaccine policies and strategies must include an ongoing process of documenting the information needs, questions, and apprehensions of their health workers and address them in real-time.

## Appendices

Appendix 

**Table 2:** Health workers: COVID-19 acceptance survey questionnaire Credits: Khurram Shahzad, Zaeem Ul Haq, and Assad Hafeez.

Code# ☐☐☐☐											
Demographics (Please write or tick where appropriate)											
Name											
Resident of											
Gender	Male ☐	Female ☐									
Age (Years)	18-30 ☐	31-50 ☐	>50 ☐								
Professional category	Doctor ☐	Nurse ☐	Lab tech ☐	Paramedic ☐		Polio worker ☐		Ancillary ☐		Other ☐	
Experience (Years)	0-10 ☐	11-20 ☐	>20 ☐								
Medical history (Please tick where appropriate)											
Confirmed COVID-19 episode	Currently +ve ☐	Covid-19 past 3 months ☐	COVID-19 before > 3 months ☐								
Co-morbidity	DM ☐	HTN ☐	IHD ☐	Cancer ☐					None ☐		
Willingness for Covid-19 vaccine (Please tick where appropriate)											
Want the vaccine	Yes ☐	No ☐									
Reasons of not yet vaccinated	AVC is too far ☐	Busy in duties ☐	Unsure re logistics ☐	Waiting efficacy data ☐		Pregnant or lactating ☐	Fears adverse effect ☐		Co-morbid condition ☐	Just had Covid-19 ☐	Other ☐
Do not want the vaccine	Yes ☐	No ☐									
Reasons of not willing to vaccinate	AVC is too far ☐	Busy in duties ☐	Unsure re logistics ☐	Waiting efficacy data ☐	Pregnant or lactating ☐		Fear adverse effects ☐		Co-morbid condition ☐	Just had Covid-19 ☐	Other ☐
Remarks if any:											
Name of data collector----------- Date ------------- Signatures data collector---------											
